# Anaerobic Biodegradation of Polylactic Acid-Based Items: A Specific Focus on Disposable Tableware Products

**DOI:** 10.3390/ma18051186

**Published:** 2025-03-06

**Authors:** Marica Falzarano, Alessandra Polettini, Raffaella Pomi, Andreina Rossi, Tatiana Zonfa, Maria Paola Bracciale, Serena Gabrielli, Fabrizio Sarasini, Jacopo Tirillò

**Affiliations:** 1 Department of Civil, Building and Environmental Engineering, Sapienza University of Rome, Via Eudossiana 18, 00184 Roma, Italy; marica.falzarano@uniroma1.it (M.F.); raffaella.pomi@uniroma1.it (R.P.); andreina.rossi@uniroma1.it (A.R.); tatiana.zonfa@uniroma1.it (T.Z.); 2 Department of Chemical Engineering Materials Environment, Sapienza University of Rome, Via Eudossiana 18, 00184 Roma, Italy; mariapaola.bracciale@uniroma1.it (M.P.B.); fabrizio.sarasini@uniroma1.it (F.S.); jacopo.tirillo@uniroma1.it (J.T.); 3 School of Science and Technology, Chemistry Division, Via Madonna delle Carceri (ChIP), University of Camerino, 62032 Camerino, Italy; serena.gabrielli@unicam.it

**Keywords:** biodegradability, bioplastics, mesophilic anaerobic digestion, polylactic acid, principal component analysis

## Abstract

The viability of anaerobic degradation treatment as an end-of-life option for commercial disposable bioplastic tableware, typically certified as compostable, was assessed. Two types of polylactic acid-based items were selected and tested under mesophilic conditions (38 °C) for 155 days, until reaching a plateau. Advanced chemical characterization of the products was performed with a combination of analytical techniques, i.e., microscopy, spectroscopy, and thermogravimetry. Two methods for calculating the biodegradation degree of the products were discussed and compared, using the biogas generated in the test and the total organic carbon (TOC) removal, respectively. The method based on TOC removal, resulting in a biodegradation degree ranging from 80.5% to 88.9%, was considered to more accurately describe the process. Given the complexity of assessing the biodegradation of a bioplastic product, an effort was made to derive correlations among the chemical–physical composition of the product, the biodegradation conditions, and the biodegradation yields/kinetics, with an aim to describe the process comprehensively. Statistical tools were also applied to derive additional considerations regarding the influence of the polymeric blend and digestion parameters on the biodegradation of bioplastic products. The identified data clusters, which were found to be grouped by the digestion temperature and the type of bioplastic, indicated specific biodegradation features of the investigated materials.

## 1. Introduction

Bioplastics have been developed over the last decades as a potential sustainable substitute for commodity plastics. Currently, biopolymers are employed in many different fields, such as medicine, ecology, and in various industrial sectors. One of the most successful applications is their use to replace packaging materials and disposable items [1,2]. On the other hand, there are still concerns about the environmental implications related to the extensive consumption of bioplastics. Key issues include their actual biodegradability in natural compartments, their limited recycling/recovery potential compared to traditional plastics, the generally scarce consumer awareness regarding discarded bioplastics’ fate, their intrinsic low durability and related short lifetime, land/resources consumption associated to their production, and the potential generation of secondary degradation products [3,4]. Polylactic acid (PLA) is an aliphatic polyester widely used for such purposes, with a reported global production capacity of 0.92 Mt in 2024 [1]. PLA is produced from lactic acid, mainly by direct polymerization or ring-opening polymerization [5], and can differ in its chemical characteristics depending on the relative quantity of D- and L-lactide, which are two stereoisomers of lactic acid [6,7]. PLA is usually distinguished by its brittleness, which generally requires a plasticization process to increase its ductility and processability. Moreover, it can be co-polymerized and blended with additives to attain the required physical and mechanical characteristics (thermal and mechanical resistance, barrier properties, transparency, etc.) [8,9]. The molecular weight and polymeric chain length, together with the chemical composition and morphology of the final blend, can strongly influence the biodegradation mechanisms of the material. PLA polymers can have different molecular weights and crystallinity degrees which can strongly affect the depolymerization process [10]. The biodegradation of PLA is known to occur in three stages [11]. The first and most limiting step in PLA degradation is the cleavage of the ester backbone, which can be attained through the combined action of chemical and enzymatic hydrolysis (with hydrolytic enzymes such as protease, esterase, lipase, and cutinase having been studied to promote the cleavage of polymer chains) [11,12]. The second step involves the fragmentation of the material into oligomers and/or monomers. It is suggested that microbial degradation can occur only when the molecular weight of PLA falls below 10,000 Da [13]. After this stage, the material can be easily mineralized by microorganisms [14].

PLA-degrading bacteria, especially mesophiles, are hardly found in natural environments [15], and only a limited number of them has been isolated as of yet [16]. In particular, the family Pseudonocardiaceae and related genera, including Amycolatopsis, Saccharothrix, Lentzea, Kibdelosporangium, and Streptoalloteichus have been recognized as PLA degraders [17]. The interest in “rare actinomycetes”, as listed above, is currently increasing as these microbes are known to be capable of hydrolyzing complex molecules [18]. Of course, the environmental conditions that the material ends up in also play a role during the process [19,20]. The degradation temperature plays a crucial role in accelerating biodegradation kinetics. As the temperature approaches the glass transition temperature, the polymeric chains become more flexible and susceptible to cleavage. However, at ambient temperatures, this process is significantly hindered, resulting in very slow PLA degradation rates [21].

In this framework, it is clear that the management of bioplastic residues should take into account the transformations that take place in a real biodegradation system, assessing the kinetics and yield associated with the biodegradation process, as well as the potential generation of intermediate or undesired by-products [22]. Treating bioplastics residues through anaerobic digestion may be beneficial since it could allow the recovery of both materials and energy, provided that bioplastics do not interfere with the typical conditions of conventional treatment. The scientific literature has begun addressing bioplastic-related issues in organic waste management only recently, and so the available information is still limited and has not yet reached indisputable conclusions [23,24].

It has been reported that biodegradation is promoted by high temperatures (not only due to thermodynamic reasons but also as a result of changes in the polymer’s properties when close to its glass transition temperature) and by low degrees of crystallinity [25]. A study that compared semi-crystalline and amorphous PLA under high-solids mesophilic conditions for 170 days reported for the latter a biodegradation level of only 36%, while the semi-crystalline polymer did not display any significant sign of biodegradation [26]. A number of authors tested PLA products under thermophilic and mesophilic conditions and found that temperature had an influence mainly on biodegradation kinetics. For instance, Vasmara and Marchetti tested PLA cups for 90 days and observed 56% degradation at 55 °C and no detectable degradation at 35 °C [27]. Experimental runs on PLA sheets at 38 and 58 °C required degradation times of 500 and 100 days, respectively, to reach a biodegradation degree of 75% [28]. Other authors, when testing PLA cups at 37 °C, reached 66% conversion in 280 days [29]. In another study treating PLA-based laboratory blends (~80% PLA content), the material could be degraded at levels of 84–100% only when thermophilic conditions (55 °C) were adopted [30]. However, even when thermophilic conditions are adopted, a significant scattering of the biodegradation degrees can be observed. This suggests that material composition has a more prominent influence on the process. Additional studies testing commercial PLA-based items at 55 °C confirm this observation. Some PLA cups reached 96% conversion in 100 days [31], and PLA cutlery and plates tested for 90 days showed biodegradation degrees of 58.4% and 78.3%, respectively [32], while some coffee capsules and packaging showed no biological conversion into biogas after 35 days [33]. A number of authors have reported that the high molecular weight and crystallinity in PLA cause slower degradation kinetics, which may be overcome with suitable chemical pre-treatments [34,35]. The hindering effect of increased crystallinity was observed in another study when comparing individual polymers with blends, which showed a lower degree of crystallinity and improved degradation kinetics [30]. Since mesophilic digestion temperatures may be more advantageous for the processing of residual bioplastics in terms of energy demand and process stability [36], it is useful to infer how bioplastic residues can be managed under mesophilic conditions and what limitations to biodegradation exist under such operating conditions.

The combination of the operating parameters of the biodegradation process and the interactions these can have with the physical and chemical properties of bioplastics are difficult to predict. The correlations among the aspects concurring in biodegradation need to be investigated thoroughly to understand how to handle bioplastic residues correctly.

In the present study, the anaerobic degradability of disposable PLA-based items was evaluated under batch mesophilic conditions for 155 days, with the aim of investigating the maximum biodegradation potential over time for two different products. Anaerobic conditions were selected to evaluate the viability of such treatment as an end-of-life option for commercial disposable bioplastic tableware, typically certified as compostable. The composition and chemical properties of the products were evaluated by combining different testing methods (physical, chemical, and microscopic analyses) to understand the influence of the matrix constituents on the biodegradation process. Mesophilic conditions were adopted to evaluate the behavior of the products within a temperature range that is widely used in full-scale applications and is critical to bioplastic biodegradation but has rarely been investigated. Different calculations for the biodegradation degree (one based on biogas production and the other on organic carbon removal) were used and discussed in order to compare multiple approaches to quantify the biodegradation process.

The same items had been also tested during a previous experimental campaign under thermophilic conditions [37], which was used here to draw some new comparisons and elaborations. In this regard, an element of innovation involved the statistical analysis of the experimental results of both experimental campaigns with the purpose of identifying the most relevant factors affecting the biodegradation features of the investigated materials. The results of this investigation can assist advanced bioplastic waste management, specifically elucidating the behavior of these residues during biological treatments for valorization. The novelty of this part of the study was the attempt to obtain in-depth insights into the biodegradability of commercially available bioplastic products as well as into the influence of the nature of bioplastic products and the digestion operating conditions on the degradation process. The current scientific literature reports variable responses to anaerobic degradation depending on the biopolymers’ properties and the digestion conditions [24]. By combining multiple analytical techniques and different biodegradation assessment methods, our study aims to provide a multidimensional evaluation of PLA degradation. From a long-term perspective, this information can be used not only for the comprehension of environmental behavior but also for the identification of the most appropriate strategies for end-of-waste bioplastic disposal and/or valorization.

## 2. Materials and Methods

### 2.1. Feedstock Materials

Two PLA-based commercial items, namely a single-use cup (PLA1) and a plate (PLA2) from the same manufacturer, were selected for the biodegradation tests. The sample selection was conducted on the basis of the following criteria: (i) wide commercial availability and abundance in organic waste streams; (ii) use as biodegradable counterparts of conventional plastics; and (iii) certified as compostable according to the UNI EN 13432:2002 standard [38].

The edges and bottom of the cups were removed, as well as the edges of the plate, since they had higher thickness and rigidity and would have affected material homogeneity. The lateral surface of the cups and the bottom of the plate were further cut into 1.5 × 1.5 cm squares. The inoculum used for the biodegradation tests was a digestate collected from an anaerobic digestion plant of agricultural residues operating at 38 °C. Microbiological assays performed on the sludge identified the predominance of acetoclastic methanogens, notably *Methanosarcina* and *Methanoculleus*, whose prevalence indicates a well-operated anaerobic digestion system. After sieving at 840 μm according to ISO standards [39,40], the digestate was stored at −15 °C to preserve its characteristics up until the start of the test. The required aliquots of the inoculum were then defrosted at room temperature before each set of tests. Commercial-grade calcium lactate (LACT; C_6_H_10_CaO_6_·5H_2_O) acquired from Special Ingredients (Savona, Italy) was used as a positive control in accordance with ISO 14853 [41] which requires that the reference material exceeds 70% biodegradation within 60 days for the test to be considered valid. The main characterization parameters (total solids (TSs), volatile solids (VSs), total organic carbon (TOC), and hydrogen) of the feedstock materials are shown in Table 1. The TS and VS concentrations were determined according to the Standard Methods for the Examination of Water and Wastewater [42]. The TOC concentration was measured using a Shimadzu TOC analyzer equipped with modules for the analysis of both liquid and solid samples (TOC-VCHS and SSM-5000 module, Shimadzu, Kyoto, Japan). The elemental hydrogen content was determined through the dry combustion method using the elemental analyzer CHN600 (Leco Instrumente GmbH, Mönchengladbach, Germany). The molecular weight distributions (MWDs) were assessed by Gel Permeation Chromatography (GPC) using Chloroform (≥99.8%, Sigma Aldrich, Saint Louis, MO, USA) eluent. The GPC measurements (Table 2) were carried out with an Agilent 1260 Infinity II Multi-Detector Suite (MDS) device (Agilent Technologies, Santa Clara, CA, USA). This device contained an Agilent 1260 Infinity Quaternary Pump (G7111B) (Agilent Technologies, Santa Clara, CA, USA), containing a 4-channel vacuum degasser to pump the eluent into the system. The autosampler was G7129A, and the thermostat column compartment was G7116A. The used device consisted of three different detectors (G7800A): a dual light scattering detector (measuring in the angles of 15° and 90°), a refractive index detector RI operating at 658 nm, and a viscosimeter VS-detector. The THF mobile phase contained 250 ppm of butylhydroxytoluene (BHT; ≥99.0%, Sigma Aldrich, Saint Louis, MO, USA), and the flow rate was fixed at 1.0 mL/min. The THF GPC system was equipped with two 5 μm columns (30 cm) in series, a mixed C and a mixed D column. A guard column (Agilent GPC/SEC Guard Column, an HPLC Guard Column PLgel 5 μm, 7.5 × 50 mm) was settled. A series of ten narrow-monodisperse polystyrene (PS) standards (Mp values ranging from 580 to 283,800 g mol^−1^; Agilent Technologies, Santa Clara, CA, USA) were used for column calibration. The bioplastic products were investigated for their chemical–physical characteristics using a combination of Scanning Electron Microscopy (SEM; Mira3, Tescan, Brno, Czech Republic), Differential Scanning Calorimetry (DSC; DSC 214 Polyma, Netzsch, Selb, Germany), thermogravimetry (TGA; Setaram Instrumentation, Caluire-et-Cuire, France), and Fourier Transform Infrared Spectroscopy (FT-IR; Bruker Optik GmbH, Ettlingen, Germany). The analytical methods used for the characterization of the feedstock materials and the related experimental results are described and discussed in detail in Bracciale et al. [37]. We just report here the main features of the materials tested that were deemed useful to interpret the experimental results. The SEM analyses revealed a relatively neat morphology for PLA1, while PLA2 displayed a homogeneous polymeric matrix containing a number of irregular inclusions. This was confirmed by FT-IR spectra evidencing the characteristic bands of the PLA polymer [43] and the presence of CaO, CaCO_3_ [44], TiO_2,_ and talc (Mg_3_Si_4_O_10_(OH)_2_) as additives. The DSC results highlighted a higher degree of crystallinity of PLA1 (χ = 37.5%), while PLA2 showed a low crystallinity degree (χ = 0.5%), despite the presence of additives which are typically known to act as nucleating agents [45,46,47]. However, crystallization can also depend on the processing conditions, molecular weight, and content of L, D, or meso-lactide in the main chain [48], which probably determined the different crystallinity degree of the two investigated materials.

**Figure 1:**
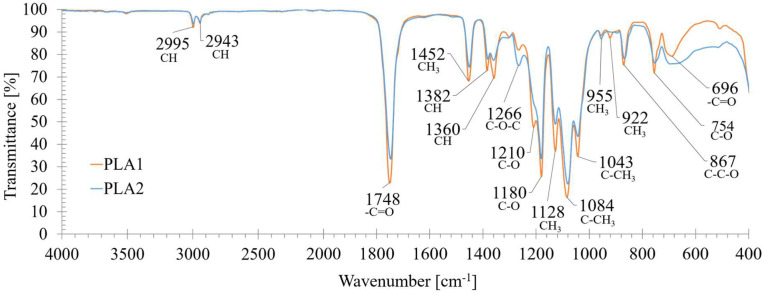
FT-IR spectra of the undigested PLA1 and PLA2 samples. The main functional groups and associated wavelengths are indicated.

FT-IR spectra (see Figure 1) showed the typical PLA absorption bands, in particular deformation and asymmetric bendings of -CH- groups at 1382 cm^−1^ and 1360 cm^−1^, respectively, the asymmetric bending of -CH_3_ at 1452 cm^−1^, the carbonyl group (-C=O) asymmetric stretching at 1748 cm^−1^ and 696 cm^−1^, and the -C-O-C stretching and CH bending modes at 1266 cm^−1^. Two additional bands at 867 cm^−1^ (C-C-O stretching) and 754 cm^−1^ (C-O bending) were associated with the amorphous and crystalline phases of PLA, respectively [49,50]. The ratios between the FT-IR intensities of the bands of C-O stretching 1210/1180 (r_1_) and of the CH_3_ rocking mode 955/922 (r_2_) were used to derive the crystallinity index [43,51], which turned out to be higher for PLA1 (~16% and ~23% higher than for PLA2, respectively). This matched the features derived from the DSC analysis, according to which the thermal degradation curves displayed a lower T_peak_ for PLA2 (361 °C) compared to PLA1 (376 °C); this suggests a different polymeric grade, or it may be related to the presence of additives, which may have decreased the material’s thermal stability, as observed by other authors [52]. 

DSC analyses from the first heating cycle are summarized in Table 3 of our previous study [37] and the thermal properties that were analyzed (T_g_ = glass transition temperature; T_cc_ = cold crystallization temperature; T_m_ = melting temperature; ΔH_cc_ = cold-crystallization enthalpy; ΔH_m_ = melting enthalpy) were used in this study for statistical analysis (see Section 3.3).

### 2.2. Anaerobic Biodegradation Tests

**Figure 2:**
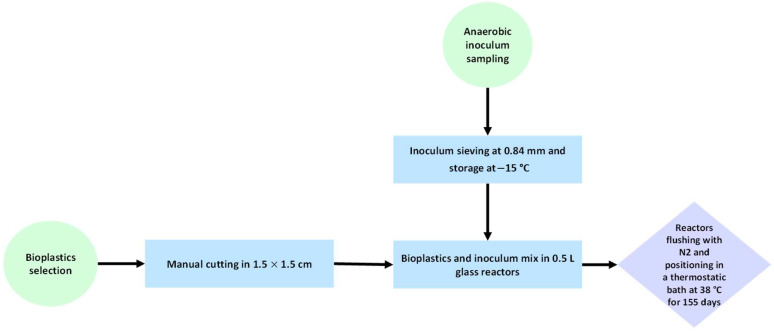
A flowchart of feedstock material preparation for the anaerobic degradation test.

The procedure adopted for sample preparation before the biodegradation tests is illustrated in Figure 2.

A Gas Endeavour^®^ (BPC Instruments, Lund, Sweden) was used for the biodegradation tests. The experimental set-up consisted of 500 mL batch airtight reactors with overhead mechanical stirring, a thermostatic bath, and a gas measurement unit integrated with a data acquisition system. The assays were carried out at 38 °C on a 400 g bioplastic + inoculum (inoculum density = 1041.6 g/L) mixture in each reactor. For PLA1, the three mixtures were tested at substrate-to-inoculum (S/I) ratios in the range commonly adopted in biomethane production potential (BMP) tests [53], namely 0.5, 1, and 2 gVS_substrate_ (gVS_inoculum_)^−1^ (see Table 3). Since the results showed that the S/I ratio played only a minor role in the material’s biodegradation (see the Section 3.1 for further details), PLA2 was only tested at a S/I ratio of 1 gVS_substrate_ (gVS_inoculum_)^−1^. Blank tests were prepared using 400 g of inoculum without bioplastics, while three additional runs with calcium lactate (LACT) at S/I ratios of 0.5, 1, and 2 gVS_substrate_ (gVS_inoculum_)^−1^ were carried out as positive control tests to assess the inoculum activity. Abiotic tests were also performed with bioplastics and deionized water (electrical resistivity > 18.3 MΩ·cm) to evaluate the degree of abiotic degradation.

For the purpose of comparing the biodegradation properties of the materials under mesophilic and thermophilic conditions, the results of a previous study on the thermophilic degradation of the same bioplastic products with the same inoculum [37] were also considered. The details of the experimental conditions tested are reported in Table 3 for completeness of information.

All tests were run in duplicate. Initial anaerobic conditions were guaranteed by flushing the reactors with N_2_ gas for 10 min at the onset of the experiments. Afterward, each reactor was connected to a 5 L gasbag, where the evolved biogas was stored. The gas was sampled once a week with a syringe and analyzed with a gas chromatograph to measure the content of H_2_, CO_2_, CH_4,_ and N_2_. The evolved biogas volume was reported within standard thermodynamic conditions (T = 273.15 K, p = 101 kPa) on the basis of the values of room temperature and pressure recorded during the tests. The incubation tests were stopped after 155 days when the daily methane production during three consecutive days was below 1% of the measured cumulated methane volume [54]. The methane volumes were derived from biogas composition analysis through gas chromatography (Model 3600 CX, VARIAN, Palo Alto, CA, USA) according to the methodology indicated in [37].

The biogas production data were interpolated through the modified Gompertz equation (Equation (1)) using TableCurve 2D v. 5.01 (Systat Software GmbH, Erkrath, Germany). The Gompertz equation was applied to estimate the kinetic parameters of the biodegradation process and extrapolate the ultimate biodegradation degree:

(1) $$P\left(t\right)=P_{m}*\exp\left\{-\exp\left[\frac{R_{m}*e}{P_{m}}\left(\lambda -t\right)+1\right]\right\}$$

where *P*(*t*) is the cumulative biogas production at time *t*, *P_m_* is the maximum biogas production, and *λ* indicates the lag phase duration. The process kinetics were also assessed by calculating the ratio (*x*%) between the evolved biogas and the maximum conversion yield and evaluating the associated length of time (*t_x_*_%_) required to reach a predetermined percentage of conversion (with *x%* = 10, 25, 50, 75, or 95%).

The biodegradation degree (BD) was first calculated from the biogas volume produced (*BD_biogas_*) using Equation (2):

(2) $${BD}_{biogas}(t)=\frac{V_{net}\left(t\right)}{V_{th}}$$

where *V_net_*(*t*) is the observed cumulated biogas production (net of blank) at time *t* and *V_th_* is the theoretical biogas production potential calculated according to the Buswell equation [55] from the material’s elemental composition. The IC content was not accounted for in this calculation due to some issues related to its measurement at the end of the mesophilic tests. This led to an underestimation of the biodegradation degree that was therefore also calculated from the observed TOC removal (*BD_TOC_*) as expressed by Equation (3):

(3) $${BD}_{TOC}\;\left(t\right)=\frac{TOC_{0}-TOC_{f}}{TOC_{0}}$$

where *TOC*_0_ is the initial amount of total organic carbon associated with bioplastics and *TOC_f_* is the measured amount of residual (net of blank) particulate organic carbon attributed to bioplastics.

The final digestates were frozen at −15 °C at the end of the test to stop biological activity until the time of the analysis. The freeze and thaw effect resulted in the pulverization of the residual plastic particles that were visible at the end of the biodegradation tests, which regrettably prevented the residual bioplastics from being separated from the digestate and used for subsequent analyses. As a result, the evaluation of the degree of physical disintegration of the material upon anaerobic digestion was no longer possible, since the bulk of the digestate matrix exerted a masking effect during the FTIR analysis.

### 2.3. Statistical Methods

The results of the digestion tests, as expressed in terms of the total duration of the biodegradation process and the degree of biodegradation, were further processed using statistical methods in order to analyze the most relevant factors involved in bioplastics biodegradation. To this aim, both the results of the present study and those from our previous investigation [37] on the same materials under thermophilic conditions were included in the analysis. In particular, principal component analysis (PCA) was applied to highlight significant similarities and differences in the experimental results so as to group homogeneous experimental data and differentiate statistically distant clusters on the basis of the observed biodegradability features. PCA is a widely used statistical technique that is applied to reduce the complexity of systems governed by multiple interrelated variables while retaining the important information conveyed by the original factors themselves. PCA is capable of identifying features, trends, and patterns in the dataset of concern, as well as grouping the results based on similarities or separating groups/individuals that show statistical differences.

Basically, linear PCA derives a set of new mutually uncorrelated (orthogonal) variables, the so-called principal components (PCs), through linear transformation of the original variables with the constraint of capturing most of the variance of the data. The PCs are thus ranked in decreasing order of the explained variance of the associated dataset.

The analyzed dataset was arranged in a (*m* × *p*) matrix, with *m* and *p* indicating the number of data points and variables, respectively. The variables included: (1) parameters related to the physical properties (thickness [th], glass transition [T_g_], cold crystallization [T_cc_] and melting [T_m_] temperatures, cold crystallization [ΔH_cc_] and melting [ΔH_m_] enthalpies, and residual weight at the end of the TGA test [w_res_]) and chemical properties (TOC and VS contents) of the tested materials; (2) biodegradation conditions (digestion temperature [T_dig_] and S/I ratio); and (3) biodegradation characteristics (t_95_ and the biodegradation degree [biod] at the end of the digestion tests), representing the response variables for the study. It is noted that the other variables analyzed in the study (see the Section 3) were not included in PCA as they were mutually correlated with those listed above, as assessed through a preliminary Spearman’s correlation screening. The data points used for the statistical analyses included those derived from both the mesophilic tests and from the previous thermophilic tests [37]. PCA was carried out using the two packages FactoMineR and FactoExtra [56,57], available in the R environment (R version 4.3.2, http://www.r-project.org/ [58]).

## 3. Results and Discussion

### 3.1. Mesophilic Anaerobic Biodegradation Tests

**Figure 3:**
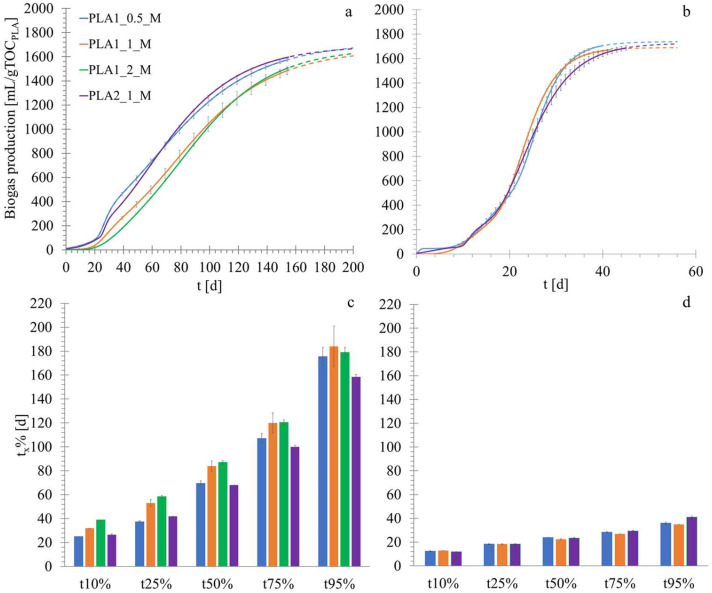
Results of the biodegradation tests: specific biogas production interpolated with the Gompertz equation (see Section 2.2) (the dashed lines indicate the extrapolated curves beyond the end of the experiments) under (**a**) mesophilic conditions and (**b**) thermophilic conditions [37]. Evolution of *t_x_%* (the time required to reach a predetermined fraction of the total biogas produced) along the biodegradation process under (**c**) mesophilic and (**d**) thermophilic conditions [37].

No significant degradation phenomena were observed during the abiotic tests, as determined upon inspection of the shape and size of the final PLA particles, indicating that no appreciable dissolution phenomena occurred as a result of the chemical hydrolysis of PLA in water. The tests with calcium lactate (positive control material) testified the adequate biological activity of the inoculum as indicated by ISO 14853, which reached 70% degradation within 50 days. Moreover, the positive control test showed no lag time in biogas production, indicating the absence of inhibiting effects of the inoculum storage method before testing, as well as of other potential limiting factors for microbial growth. The specific biogas yield (a) and the *t_x_*_%_ parameter are reported in Figure 3. The tests lasted 155 days, the time at which they were terminated on the basis of the residual methane production observed. Some bioplastic fragments were visually identifiable at the end of the biodegradation tests, although it was not possible to evaluate the degree of material disintegration (see the Section 2.2). The specific cumulative biogas production ranged between 1665 and 1736 mL (gTOC_PLA_)^−1^ (corresponding to a net production of 1163–1404 mL gTOC_PLA_^−1^) for all the experimental runs (Figure 3a), while the methane production ranged between 831 and 1251 mL (gTOC_PLA_)^−1^ (a net production of 746–912 mL (gTOC_PLA_)^−1^. For PLA1, shifting the S/I ratio from the value of 1 gVS_substrate_ (gVS_inoculum_)^−1^, commonly adopted in BMP tests [53], produced only a relatively small variation (−9% at S/I = 0.5 and +9% at S/I = 2) in the extent of organic carbon conversion into biogas. The effect of the S/I ratio on the biodegradation kinetics was different at S/I = 0.5 and S/I = 2. In the latter case, a reduction in the biodegradation process kinetics was observed, with t_10%_ and t_25%_ being 22% and 10%, comparatively higher than at S/I = 1 (Figure 3b).

However, the differences in the degradation rate among the PLA1 samples at the three S/I ratios tended to decrease gradually as biodegradation proceeded, so that the t_95_ value was similar regardless of the S/I ratio. This could suggest that the hydrolysis was faster at lower initial substrate amounts. A similar result was obtained by Cazaudehore et al. [59] when testing PLA and PHB, who observed enhanced kinetics when progressively decreasing the S/I ratio from 0.5 to 0.1.

On account of the relatively slight differences in both the final conversion yield and the total biodegradation duration at different S/I ratios for PLA1 (cup), the PLA2 sample (plate) was only tested at S/I = 1. By comparison of the two products, it was observed that the rate of PLA2 degradation was higher than PLA1 (17–21% faster in the initial process stages and 14–17% faster in the final stages) (Figure 3b). This effect was related to differences in the chemical or physical properties of the two items, including the higher crystallinity of PLA1 that may have slowed down the biodegradation process. The literature results showed a crystallinity degree increase during the first stages of the PLA biodegradation process [15], which is correlated to the faster erosion of the amorphous regions [35]. Moreover, it has been reported that the high crystallinity of PLA hinders molecular weight reduction [60,61].

The biodegradation degree (see Table 4) was in the ranges of 62.4–73.4% and 80.5–88.9% when calculated according to Equation (2) (*BD_biogas_*) and Equation (3) (*BD_TOC_*), respectively. The former range represents an underestimation of the biodegradation degree since it does not account for the dissolved fraction of CO_2_ (see Section 2.2), while the latter is considered to be more representative of the real degradation degree achieved. This shows that the amount of CO_2_ dissolved in the liquid phase provides a non-negligible contribution to the estimation of the degree of biodegradation through the measurement of the evolved gas. The behavior of PLA1 and PLA2 in terms of overall biodegradability was found to be substantially similar, with PLA2 displaying 6.8% (*BD_biogas_*) and 4.7% (*BD_TOC_*) higher yields compared to PLA1. Neither of the two samples reached complete biodegradation by the end of the anaerobic digestion tests (155 days), indicating that 12.2–19.5% (PLA1) and 11.1% (PLA2) of the initial bioplastic material remained as an undegraded residue in the final digestate.

### 3.2. Comparison of Mesophilic and Previous Thermophilic Experimental Campaigns

As indicated in Section 2.2, the same PLA products tested in the present study had also been previously investigated under thermophilic anaerobic conditions. For detailed results, the reader is referred to our previous publication [37]; part of the experimental data was used here (in some cases after some reprocessing to make it consistent with the rest of the dataset) for comparison purposes in order to highlight the influence of the digestion temperature on biodegradation. A summary of the main results obtained in terms of final biogas production and the final biodegradation degree is reported in Table 4.

The most evident difference observed between mesophilic and thermophilic digestion involved the biodegradation kinetics, and the time required to reach the same final biogas production was almost fivefold shorter under thermophilic conditions. Of course, higher temperatures play a role in increasing both the mobility of the polymeric chain and the enzymatic activity, therefore reducing the polymer hydrolysis time [12]. Moreover, while under thermophilic conditions the biodegradation degree attained was comparable for all the tests (Table 4); the PLA2 degradation kinetics were found to be 15% slower than for PLA1, which was attributed to the higher complexity of the polymeric matrix [37]. When compared to the thermophilic regime, the mesophilic process highlighted some criticalities for PLA1, since the degradation rate not only slowed down but also became slower than for PLA2, and at the same time the attained degree of biodegradation (BD_TOC_) was 6–12% lower compared to thermophilic conditions. Both these aspects were related to the higher crystallinity of PLA1, which was clearly a limiting factor under mesophilic conditions. This effect is of course mitigated at thermophilic temperatures which are closer to the T_g_ of the material, thus increasing flexibility and liability to hydrolysis [36,62].

On the other hand, no effects were observed on the degradation degree of PLA2 when the temperature was decreased, for which the extent of degradation was more likely governed by the matrix composition (see Figure 3). While the S/I ratio only exerted a slight impact on the biodegradation process under mesophilic conditions, this was even less evident under thermophilic conditions. This suggests that the interactions among the operating parameters and the polymeric blends are complex and must be investigated in detail, especially when adopting mesophilic temperatures that amplify the effects of such factors.

Another distinguishing feature of mesophilic and thermophilic conditions involved the presence of residual undegraded bioplastic particles at the end of the digestion tests. While, as noted in Section 3.2, some bioplastic fragments were visually identifiable at the end of the mesophilic tests (indicating that the biodegradation process was not completed under such conditions), disintegration at thermophilic temperatures was found to be complete [37].

A number of studies report no biodegradation for commercial PLA products (cups, coffee capsules, and packaging) both under mesophilic (35 °C, 90 days) and thermophilic (55 °C, 35 days) conditions [27,33] The same issue concerns the bioplastic bags currently used for the collection of the organic fraction of waste. For instance, some PLA/PBAT bags tested at 37 °C displayed a mass loss in the range of 3–25% [63]. When using prolonged retention times, a 66% biodegradation degree was attained in 280 days for commercial PLA cups tested under mesophilic anaerobic conditions [29]. In the present study, an almost complete biodegradation was attained in 155 days. This clearly indicates that the material composition strongly affects the degradation process, and the heterogeneity and presence of recalcitrant components can hinder the mineralization process. However, so far it is not possible to derive indisputable conclusions from the correlations among operating conditions, product composition, and biodegradation degree [24,25], emphasizing the need for further systematic assessments of bioplastics degradation.

### 3.3. Statistical Analysis of Parameter Effects on Biodegradation

**Figure 4:**
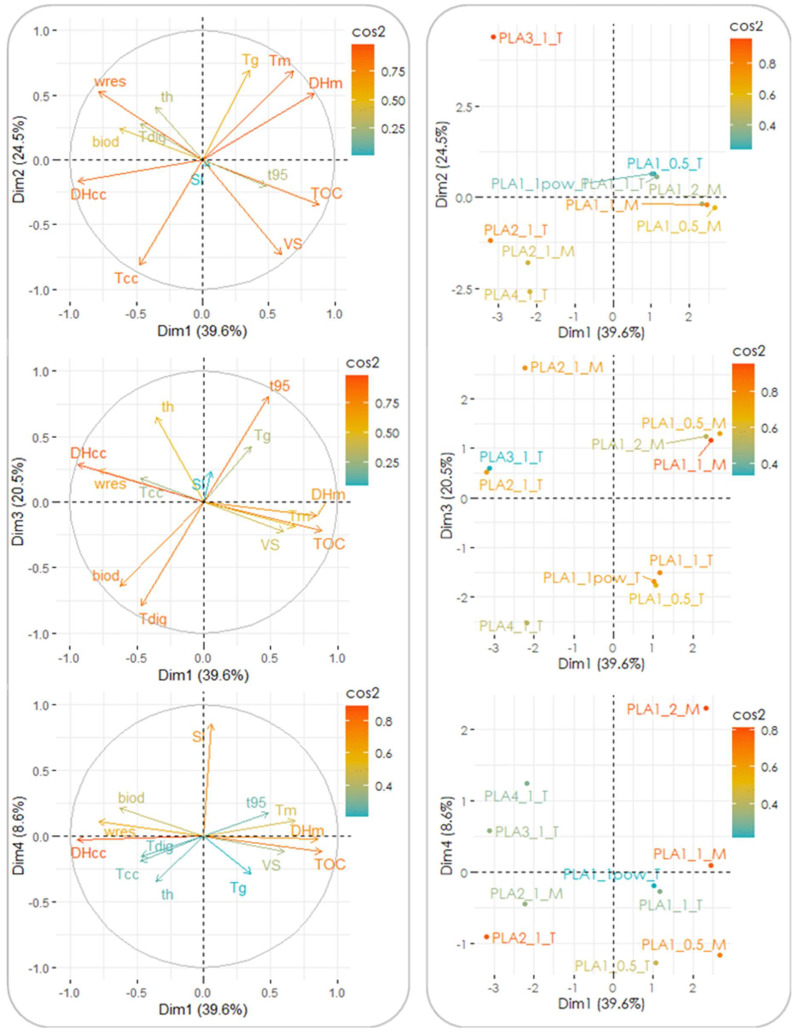
Loading plots (**left**) and score plots (**right**) of the experimental data in the first 4 PCs’ space. The loading plots depict the original variables as vectors on the PC plane (with the vector direction and module showing the correlation with each PC), while score plots depict the individual data on the same plane (visualizing the potential grouping of data). Acronyms used for the variables are as follows: thickness = th; glass transition temperature = T_g_, cold crystallization temperature = T_cc_; melting temperature = T_m_, cold crystallization enthalpy = ΔH_cc_; melting enthalpy = ΔH_m_, residual weight at the end of the TGA test = w_res_; TOC content = TOC; VS content = VS; digestion temperature = T_dig_; S/I ratio = SI; time to reach 95% of maximum biogas production = t_95_; biodegradation degree = biod.

Further attempts at identifying the influence of the tested parameters on the biodegradation features of the two bioplastic products were made by means of statistical analysis. This was aimed at finding the relevant parameters for the biodegradation process, identifying sets of data displaying similarities, and distinguishing between clusters with evident statistical differences. The analysis of the experimental data (including the results of both the mesophilic and thermophilic tests), performed through PCA, revealed that the first four principal components (PCs) accounted for ∼93% of the overall variance (although the first three explained 85% of it already); therefore, the contribution of the remaining factors to the data variability was considered to be negligible. In the loading plots reported in Figure 4, the original variables are plotted in the plane of each PC couple as vectors, the direction and magnitude of which are a measure of their correlation with the PCs. In particular, larger magnitudes and lower angular displacements of the vectors from a given axis indicate a higher correlation with the PC plotted along that axis. Furthermore, variables lying on the same quadrant are positively correlated (or negatively correlated when lying on opposite quadrants), while orthogonal variables are interpreted as being uncorrelated. The estimated correlations between the PCs and the original factors resulted in the following relationships (only the cases displaying correlations ≥0.65 are reported): (i) PC_1_ was correlated to ΔH_cc_ (r = −0.94), TOC content (0.88), ΔH_m_ (0.85), w_res_ (−0.78), and T_m_ (0.68); (ii) PC_2_ was correlated to T_cc_ (−0.81), VS content (−0.73), T_g_ (0.69), and T_m_ (0.68); (iii) PC_3_ was correlated to t_95_ (0.81), T_dig_ (−0.79), thickness (0.65), and biodegradation degree (−0.65); and (iv) PC_4_ was correlated to the S/I ratio (0.85). It is noted that the response variables of the degradation tests (t_95_ and biodegradation degree) were both associated with PC_3_ and also displayed a mutual negative correlation (r = −0.86), clearly showing that increased biodegradation was also accompanied by enhanced rates of bioplastic conversion. On the other hand, the physical, thermodynamic, and chemical properties of the investigated bioplastic products were included in PC_1_ and PC_2_, while the digestion conditions were mainly accounted for in PC_3_ and PC_4_.

It was interesting to note that, while the chemical properties of the bioplastic products were mutually correlated as visible in Figure 4, no evident correlation was found to exist between the biodegradation parameters (biodegradation degree and t_95_) and such chemical properties. This may be interpreted in the sense that the process was dominated mainly by the temperature regime adopted, which masked the influence of the other chemical properties. In addition, the influence of the bioplastic type on biodegradation was evidenced by the cluster analysis conducted (see below for further details).

It is clear that the thermodynamic and physical properties of the tested materials play a relevant role in determining their actual biodegradation features, which also explains the large variability of the results in terms of the biodegradability commonly reported in the scientific literature on different bioplastic products, including PLA-based items [23,24].

**Figure 5:**
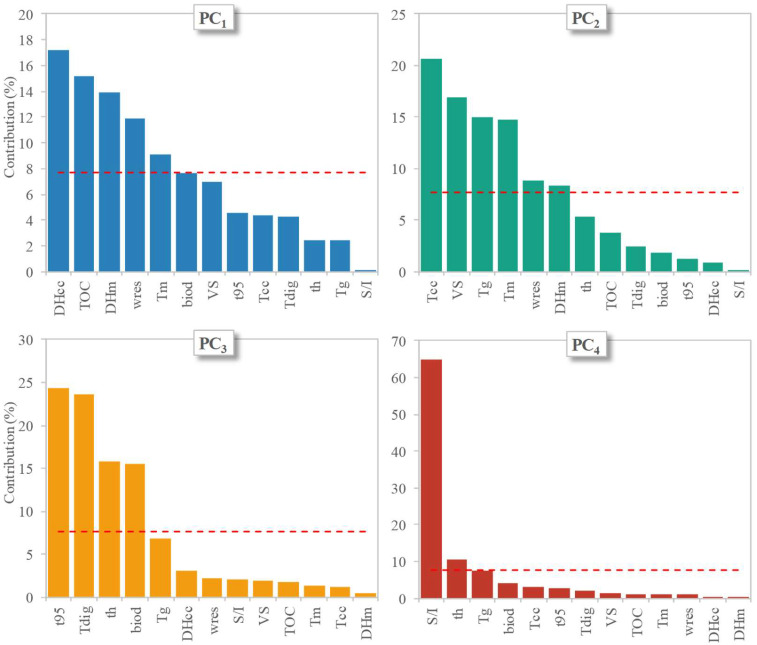
The individual contributions (in percent) of the original variables, showing their importance to each of the first 4 PCs. The horizontal dashed lines show the level expected if all 13 variables displayed the same contribution (100/13 × 100 = 7.7%) to each PC. Acronyms used for the variables are as follows: thickness = th; glass transition temperature = T_g_, cold crystallization temperature = T_cc_; melting temperature = T_m_, cold crystallization enthalpy = ΔH_cc_; melting enthalpy = ΔH_m_, residual weight at the end of the TGA test = w_res_; TOC content = TOC; VS content = VS; digestion temperature = Tdig; S/I ratio = SI; time to reach 95% of maximum biogas production = t_95_; biodegradation degree = biod.

The individual contribution of the original variables to each PC is depicted in Figure 5, in which the horizontal line represents the average value that would be observed if all variables contributed equally to the PC of concern (in this case = 100%/13 = 7.7%).

In particular, the most significant contributions (i.e., above the average) to the four PCs were found to be given by the following: (i) ΔH_cc_, TOC content, ΔH_m_, w_res_, and T_m_ for PC_1_; (ii) T_cc_, VS content, T_g_, T_m_, and w_res_ for PC_2_; (iii) t_95_, T_dig_, thickness, and the biodegradation degree for PC_3_; and (iv) the S/I ratio for PC_4_. This indicates that the different PCs were capable of capturing different types of information associated with the properties of the materials tested and their biodegradability. The effects of the different characteristics of the bioplastic samples investigated and of the digestion conditions can visually be identified in the score plots shown at the right end side of Figure 4, in which the experiments (i.e., the individuals) are plotted against each couple of PCs. Notably, there was a clear distinction among the different experiments in the score plots, which could be grouped into five clusters that were distinguished first on the basis of the temperature regime of the biodegradation tests (one cluster including the mesophilic tests and a second cluster including the thermophilic tests) and further on the basis of the type of bioplastic product (the cup [PLA1] or the plate [PLA2]) (one cluster for each type of item studied). Interestingly, no grouping was found to be governed by criteria related to the S/I ratio, further confirming that this parameter—at least in the investigated range of values—played only a minor role in determining the extent and rate of biodegradation of the two bioplastic products.

From the PCA results it is therefore evident that the type of bioplastic product of concern, which in turn implies a different polymeric blend including a variety of additives and different technical properties, reflects directly on the biodegradation of the material.

## 4. Conclusions

Two commercial PLA-based disposable items were tested under anaerobic mesophilic conditions. The characterization of the as-received products by coupling various analytical techniques outlined the different compositions of the materials in terms of the crystallinity grade and the presence of additives. Such analyses aided the understanding of the differences in the degradation behavior of the investigated materials. The final biogas yield ranged between 1665 and 1721 mL (gTOC_PLA_)^−1^, with an average methane production of 851.2 ± 54.7 mL (gTOC_PLA_)^−1^ in 155 days.

The process kinetics were weakly affected by the S/I ratio, at least within the investigated range of values. A more prominent effect was exerted by material crystallinity which correlated with a decrease in the degradation rate.

The results of this study agree with the available literature results. The comparison with the results of previous thermophilic experiments to derive new elaborations and insights indicated that the lower temperature increased the degradation time by 5 times, while the final biodegradation degree was on average 9% lower for PLA1 and comparable for PLA2.

The statistical analysis of the experimental data indicated that four main principal components may be used to explain most of the changes in the results as a function of the relevant variables. While two of these components mainly included the physical, thermodynamic, and chemical properties of the bioplastics, the other two explained the main features of the biodegradation process (biogas production yield and the degradation time) and the biodegradation conditions. The cluster analysis highlighted a clear distinction in the biodegradability of the bioplastic materials depending on the type of product tested (whether the cup or the plate).

The findings of this study emphasize the need for a systematic assessment of bioplastics degradation as a function of both the material’s properties and the operating parameters of the process. As far as the implications of the study are concerned, the prolonged biodegradation time observed under batch mesophilic conditions may represent a serious issue at full scale when the anaerobic digestion of bioplastic residues is made in co-digestion with food waste or biowaste. As a matter of fact, the degree of degradation attained (>80%) indicates that residual PLA-based products have the potential to be digested at relatively low temperatures, but at the expense of long treatment durations (>5 months). Since such durations are not compatible with the residence times commonly adopted in full-scale digesters that process organic waste, the full biodegradation of PLA products at mesophilic temperatures would require dedicated pre-treatment to enhance the hydrolysis of the polymeric matrix and increase the degradation rate.

## Figures and Tables

**Table 1:** Main characterization parameters of PLA and inoculum (see table legend for further details).

Material	Type of Item	Thickness [μm]	TS ^(1)^ [%ww]	VS ^(2)^ [%ww]	TOC ^(3)^ [gC (kgTS)^−1^]	Hydrogen ^(4)^ [gH (kgTS)^−1^]	ThCH_4_ ^(5)^ [ml (gTOC)^−1^]	ThCO_2_ ^(5)^ [ml (gTOC)^−1^]	χ ^(6)^ [%]
Inoculum	---	---	6.4 ± 0.0	4.2 ± 0.0	29.1 ± 2.4	n.a. ^(7)^	---	---	---
PLA1	Cup	205	99.6 ± 0.0	99.6 ± 0.0	530 ± 1.4	55.4 ± 0.1	952.8	915	37.5
PLA2	Plate	257	99.6 ± 0.0	97 ± 0.0	484.4 ± 0.2	54.1 ± 0.2	913.4	954	0.5
LACT	---	---	77.5 ± 0.3	42.3 ± 0.3	317.9 ± 1.1	45.8 ± 0.7	933.9	933.9	---

^(1)^ Total solids. ^(2)^ Volatile solids. ^(3)^ Total organic carbon. ^(4)^ Hydrogen. ^(5)^ Theoretical production of CH_4_ (ThCH_4_) and CO_2_ (ThCO_2_), estimated from elemental analysis applying the Buswell equation (see Section 2.2) under the assumption of full conversion of the material into the final products under anaerobic conditions. ^(6)^ Crystallinity degree. ^(7)^ not analyzed.

**Table 2:** Molecular weight distributions of the undigested PLA samples measured with Gel Permeation Chromatography: number average molecular weight (Mn), weight average molecular weight (Mw), and polydispersity index (PDI).

Material	Mn [Da]	Mw [Da]	PDI
PLA1	81,500	165,200	2.028
PLA2	66,200	145,000	2.204

**Table 3:** The experimental design of this study and previous thermophilic tests used for comparison purposes. Test codes refer to the substrate type, the substrate-to-inoculum (S/I) ratio (or abiotic conditions), and the temperature regime. The bioplastic size was 1.5 × 1.5 cm for all the experimental runs, with the exception of a sample denoted with “pow” that was powdered (<0.1 cm) prior to the anaerobic degradation test.

Run	Material Size [cm]	S/I Ratio [gVS_substrate_ (gVS_inoculum_)^−1^]	Reference
PLA1_0.5_M	1.5 × 1.5	0.5	This study
PLA1_1_M		1	
PLA1_2_M		2	
PLA2_1_M		1	
PLA1_ABIO_M		--- (abiotic)	
PLA2_ABIO_M		--- (abiotic)	
LACT_0.5_M	---	0.5	This study
LACT_1_M		1	
LACT_2_M		2	
PLA1_0.5_T	1.5 × 1.5	0.5	[37]
PLA1_1_T	1.5 × 1.5	1	
PLA1_1pow_T	<0.1	1	
PLA2_1_T	1.5 × 1.5	1	
PLA3_1_T	1.5 × 1.5	1	
PLA4_1_T	1.5 × 1.5	1	

Legend: PLA1 = cup; PLA2 = plate; LACT = calcium lactate (reference material). S/I ratio adopted = 0.5, 1, or 2 gVS_substrate_ (gVS_inoculum_)^−1^. ABIO = abiotic test (bioplastics + deionized water only). M = mesophilic (38 °C); T = thermophilic (55 °C).

**Table 4:** Measured biogas production (net of blank, *V_net_*) and biodegradation degree (average value ± standard deviation) at the end of the mesophilic (38 °C) and thermophilic (55 °C) biodegradation tests. *BD_biogas_* and *BD_TOC_* are the biodegradation degrees calculated according to Equations (2) and (3), respectively (see Section 2.2).

Run	*V_net_* [mL (gTOC_PLA_)^−1^]		*BD_biogas_* [%]		*BD_TOC_* [%]	
	38 °C ^a^	55 °C ^b^	38 °C ^a^	55 °C ^b^	38 °C ^a^	55 °C ^b^
PLA1_0.5	1162.9 ± 13.8	1654.8 ± 1.6	62.4 ± 0.7	93.1 ± 0.1	80.5 ± 1.3	91.7 ± 0.4
PLA1_1	1281.0 ± 32.5	1638.8 ± 7.9	68.7 ± 1.7	89.1 ± 0.4	84.9 ± 0.6	90.7 ± 0.3
PLA1_2	1404.1 ± 6.5	---	75.2 ± 0.3	---	87.8 ± 0.3	---
PLA2_1	1369.8 ± 2.7	1659.1 ± 24.8	73.4 ± 0.1	90.3 ± 1.3	88.9 ± 0.1	89.9 ± 0.0

^a^ This study. ^b^ Bracciale et al. [37].

## Data Availability

The original contributions presented in this study are included in the article. Further inquiries can be directed to the corresponding author.
